# Ionic Liquids as Surfactants for Layered Double Hydroxide Fillers: Effect on the Final Properties of Poly(Butylene Adipate-*Co*-Terephthalate)

**DOI:** 10.3390/nano7100297

**Published:** 2017-09-28

**Authors:** Sébastien Livi, Luanda Chaves Lins, Jakub Peter, Hynek Benes, Jana Kredatusova, Ricardo K. Donato, Sébastien Pruvost

**Affiliations:** 1 Univ Lyon, INSA Lyon, UMR CNRS 5223, IMP Ingénierie des Matériaux Polymères, F-69621 Villeurbanne, France; sebastien.pruvost@insa-lyon.fr; 2 Institute of Macromolecular Chemistry AS CR, v.v.i., Heyrovsky Sq. 2, 162 06 Prague 6, Czech Republic; peter@imc.cas.cz (J.P.); benesh@imc.cas.cz (H.B.); kredatusova@imc.cas.cz (J.K.); 3 MackGraphe–Graphene and Nanomaterials Research Center, Mackenzie Presbyterian University, Rua da Consolação 896, São Paulo 01302-907, Brazil; ricardo.donato@mackenzie.br

**Keywords:** ionic liquids, poly(butylene adipate-*co*-terephthalate), layered double hydroxide, nanocomposites

## Abstract

In this work, phosphonium ionic liquids (ILs) based on tetra-alkylphosphonium cations combined with carboxylate, phosphate and phosphinate anions, were used for organic modification of layered double hydroxide (LDH). Two different amounts (2 and 5 wt %) of the organically modified LDHs were mixed with poly(butylene adipate-*co*-terephthalate) (PBAT) matrix by melt extrusion. All prepared PBAT/IL-modified-LDH composites exhibited increased mechanical properties (20–50% Young’s modulus increase), decreased water vapor permeability (30–50% permeability coefficient reduction), and slight decreased crystallinity (10–30%) compared to the neat PBAT.

## 1. Introduction

In the world of polymer nanocomposites, the continual challenge is to develop high-performance materials at a low cost [1,2,3,4,5]. Thus, polymer nanocomposites based on layered silicates, such as montmorillonite (MMT) or layered double hydroxide (LDH), have long received attention from academic and industrial research. Consequently, many studies have reported the use of organically modified clays in biodegradable, natural or biosourced matrices, such as polylactide (PLA), poly(3-caprolactone) (PCL), poly(alkyl succinates), and poly(butylene adipate-*co*-terephtalate) (PBAT), in order to produce polymer materials having excellent thermal stability, good mechanical properties as well as good water vapor and gas barrier properties for compostable films, food packaging or tissue engineering applications [6,7,8,9,10,11,12]. For example, for compostable film applications, different composites based on PBAT and different lignocellulose fibers (pupunha, munguba, kenaf fibers) were studied to increase the performances of this matrix [13,14,15]. Several surface treatment pathways have been studied: (i) grafting of organosilanes [16,17], (ii) cationic exchange [18,19,20], and (iii) anionic exchange [21,22]. A vast number of LDH modification methods are available [23], where calcination/rehydration is often a suitable approach. In this case, anionic exchange usually requires two steps composed of an initial calcination step followed by the counter anion intercalation within a solvent medium, where various conventional anions, surfactant adsorbents, and active pharmaceutical ingredients have already been used [24]. 

Recently, ionic liquids (ILs), which are ionocovalent based organic molecules presenting low melting temperature (≤100 °C) and composed of ion pairs, have been used in the field of nanocomposites as a surfactant, interfacial agent or as dispersant of nanoparticles [25,26,27,28]. They present numerous advantages such as excellent thermal stability, negligible vapor pressure, and good affinity with organic and/or inorganic materials [25,26]. They present especially good affinity to silicates, allowing dramatic morphology changes when in situ applied to sensible processes such as the Sol-Gel, mainly when applied to its first hydrolytic step [29]. Thus, various authors have studied them as alternatives to the conventional ammonium salts well-known to have low thermal stability (<180 °C), which limits their use in polymer matrices requiring high curing or processing temperatures; e.g. to obtain the best of their mechanical properties epoxy-silica nanocomposites need a strong interphase control, thus an interphase agent is necessary, but they are often post-cured at temperatures above their glass transition, demanding agents that handle long-term exposition to high temperature [30]. The ILs not only resist the curing process without degrading but also allow morphology and mechanical properties tuning, and shape memory effect, which was demonstrated to be strongly influenced by the intimate IL-silica interaction even in such complex systems [27]. These effects were also shown to extend to layered silicates, e.g., Livi et al. demonstrated that the imidazolium and phosphonium ILs play a dual role as surfactant and compatibilizing agent of layered silicates between the polymer matrix and the nanoparticle [19,26]. Thus, they highlighted a good dispersion of the treated-MMT as well as an improvement of the thermal and mechanical properties in high density polyethylene (HDPE) and poly(vinylidene fluoride) (PVDF) matrices [25,26]. More recently, various authors used ILs as surfactant agents of LDHs leading to polymer materials with enhanced properties [9,31]. Bugatti et al. developed PLA films with excellent water barrier properties for food packaging applications [31]. Later, Kredatusova et al. developed a new, fast and environmentally-friendly process based on microwave irradiation leading to an exfoliation of LDH modified with ILs in PCL matrix [9]. Nevertheless, few works have been reported in the literature on the contribution of these fillers to the polymer matrices.

Due especially to their thermal/chemical stability and interphase adhesion properties, ILs are perfectly suitable to substitute traditional dispersion/stabilization agents in polymer nanocomposites, especially in melting processes, helping to migrate the filler properties to the polymer matrix and avoiding losses by evaporation and degradation processes. Thus, in this work, new LDHs modified with phosphonium ILs were prepared and characterized by thermogravimetric analysis (TGA) and X-ray diffraction (XRD) in order to prove the intercalation of the phosphinate, carboxylate and phosphate counter anions into LDH layers. Then, small amounts (2 and 5 wt %) of these treated-LDHs were introduced into a biodegradable matrix (PBAT), forming nanocomposites where their morphologies as well as their thermal, mechanical and barrier properties were investigated.

## 2. Results and Discussion

### 2.1. Characterization of Ionic Liquids 

#### Thermal Behavior

**Figure 1:**
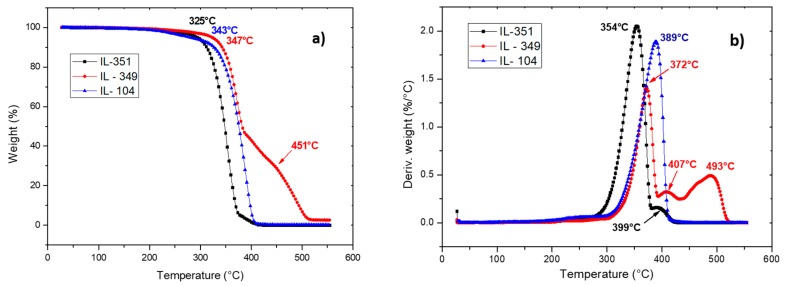
Evolution of weight loss as a function of temperature (thermogravimetric analysis (TGA) (**a**); (**b**) derivative thermo-gravimetric (DTG)) of pure ionic liquids (ILs). (heating rate 20 K·min^−1^, under nitrogen flow).

After anionic exchange with phosphonium ILs containing hexanoate, phosphinate, and phosphate anions denoted IL351, IL104, and IL349, the thermal stability of the pure ILs were investigated by TGA (Figure 1).

All the ILs presented excellent thermal stability (>300 °C), nevertheless, two behaviors are observed in Figure 1. In fact, similar degradation temperatures of the ILs denoted IL 351 and IL 104 of about 340 °C and 350 °C were obtained, respectively. In the case of IL349, three degradation peaks at 372 °C, 407 °C, and 493 °C were observed. This higher degradation temperature can be attributed to the presence of the phosphate anion. According to the literature, the phosphate compounds are commonly used as flame retardants leading to significant inflammability and a better thermal stability [32,33]. Thus, we can assume that the phosphate anion delayed the degradation of IL349 which also explains the presence of residues after 550 °C compared to the other ILs. In summary, these different ILs can be used as more thermal resistant alternatives to the thermally unstable ammonium salts, with stronger potential for polymer nanocomposites processing applications at high temperatures [34,35].

According to the literature, it is well known that pristine LDH have three degradation steps corresponding to: (i) the loss of physisorbed and intercalated water between LDH layers, which takes place between 50 °C and 250 °C [36,37,38], and (ii) the removal of interlayer carbonate anion, and (iii) dehydroxylation of –OH groups which is between 250 °C and 500 °C [39,40]. In our previous work on the surface treatment of LDH by these three ionic liquids, the presence of hexanoate, phosphinate, and phosphate anions as well as the presence of carbonate anion was also proved where characterization techniques such as TGA, Fourier transform infrared spectroscopy (FTIR) and X-ray photoelectron spectroscopy (XPS) were used. Thus, Kredatusova et al. highlighted that during the surface treatment, the regeneration of the crystalline structure of LDH induced the absorption of carbonate anions [9,38,41,42,43]. 

Based on these previous results, the influence of the surface treatment of the LDHs on the final properties of the PBAT matrix is studied hereafter. 

### 2.2. Characterization of PBAT/Modified-LDH Nanocomposites

#### 2.2.1. Morphology

**Figure 2:**
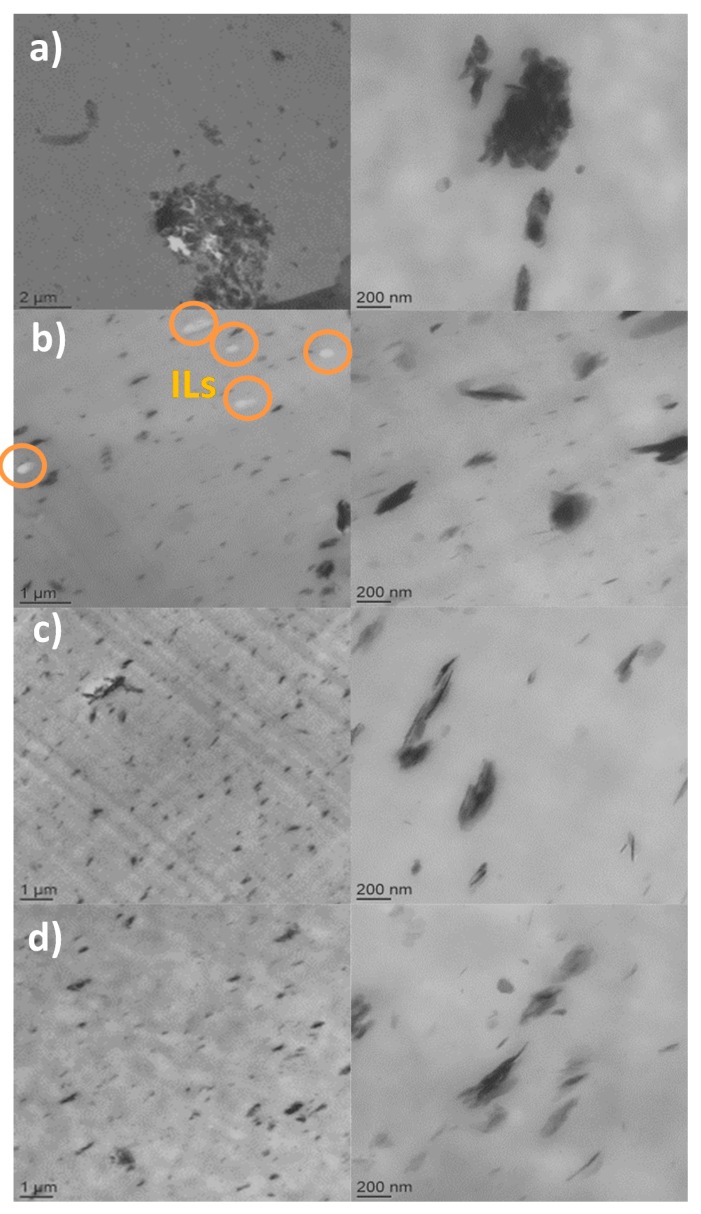
Transmission electron microscopy (TEM) micrographs of poly(butylene adipate-*co*-terephthalate) (PBAT) nanocomposites with 5 wt % of treated-LDHs: (**a**) PBAT/LDH; (**b**) PBAT/LDH-104; (**c**) PBAT/LDH-351; (**d**) PBAT/LDH-349.

**Figure 3:**
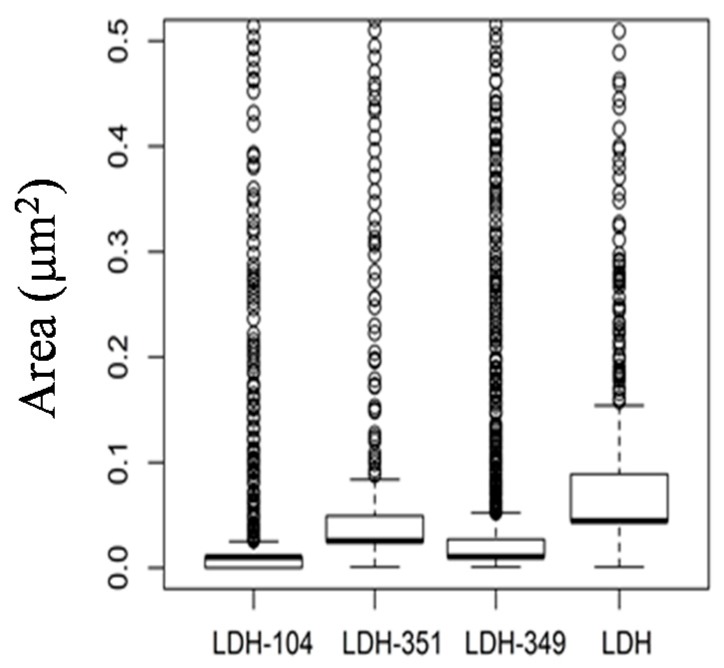
Box and whiskers plot area distribution of untreated and treated layered double hydroxides (LDHs) into PBAT matrix.

The influence of the counter anions, i.e., hexanoate, phosphinate and phosphate on the morphologies of PBAT nanocomposites containing only 5 wt % of LDH-ILs was investigated. Thus, transmission electron microscopy (TEM) micrographs and the distribution area of PBAT filled with LDH-351, LDH-104, and LDH-349 are shown in Figure 2 and Figure 3. 

In the case of PBAT filled with unmodified LDH, a very poor dispersion of LDH layers is obtained showing the presence of numerous aggregates of several microns. These results have been often reported in the literature concerning polymer nanocomposites containing untreated inorganic fillers, such as layered silicates, especially montmorillonite (MMT) [44,45].

On the other hand, a good distribution of the phosphonium IL-modified LDHs denoted LDH-351, LDH-104, and LDH-349 was observed. In fact, TEM micrographs with one micrometer scale revealed a homogeneous dispersion of LDH-ILs into PBAT matrix with the presence of a few tactoïds having sizes at the most of the order of 1–2 μm. In order to determine more precisely the type of morphology obtained, enlargements were carried out. As can be seen in Figure 2c,d, the addition of 5 wt % of LDH-351 and LDH-349 led to a mixed morphology, composed of small tactoïds and few well-dispersed clay layers in the PBAT matrix. In the case of LDH-104, an excellent dispersion is highlighted by TEM micrographs corresponding to an intercalated/partially exfoliated morphology characterized by the presence of well-dispersed clay layers combined with very small tactoïds. In addition, the presence of free IL104 defined by white dots (Figure 2b) is highlighted on the TEM images. From a previous paper on PBAT/IL blends, our research group demonstrated that the incorporation of IL104 induced a phase separated morphology due to dipole-dipole interactions between the ion pairs leading to the formation of these ionic clusters [46]. Thus, TEM micrographs confirm the presence of physisorbed IL on the LDH surface which then diffuses into the PBAT matrix during the processing of the nanocomposites by extrusion. However, the chemical nature of the counter anion plays a key role on the miscibility of ILs in polymer matrix which may explain the impossibility to observe the presence of free IL349 and IL351. 

Finally, in order to determine the level of dispersion of the untreated and treated LDHs into PBAT matrix, image analysis was carried out. Thus, the area of distribution is presented in the form of a box and whiskers plot (Figure 3). 

Independently of the system studied, image analyses are in agreement with TEM micrographs highlighting the poor area distribution of untreated LDHs (0.091 μm^2^) compared to IL treated ones. In addition, smaller distribution areas are obtained for LDH-349 (0.027 μm^2^) and LDH-104 (0.011 μm^2^) corresponding to a finer and more homogeneous dispersion of these modified LDHs. 

In conclusion, the surface treatment of the LDH by using phosphonium ILs as interfacial agents induced a good distribution of LDH-ILs in the PBAT matrix by using an extrusion process. 

#### 2.2.2. Thermal Behavior

**Figure 4:**
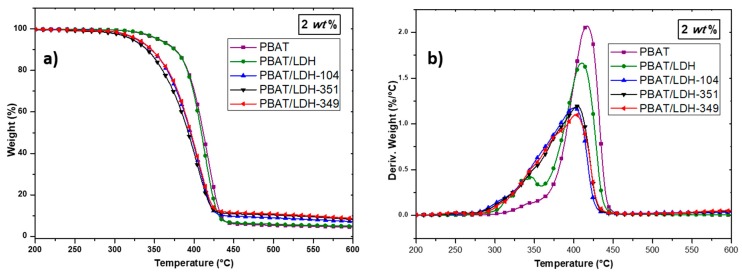
Evolution of weight loss obtained by TGA (**a**) and DTG (**b**) of the neat PBAT and PBAT-LDH (2 wt %) nanocomposites (heating rate 20 K·min^−1^ under nitrogen flow).

**Figure 5:**
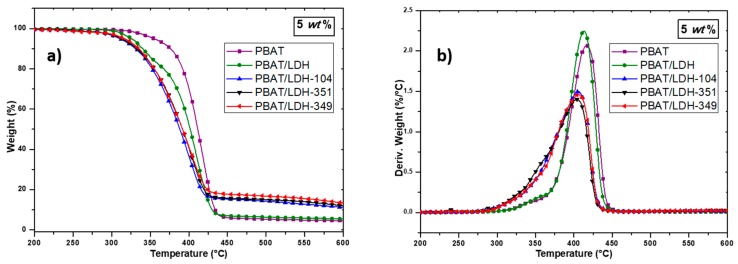
Evolution of weight loss obtained by TGA (**a**) and DTG (**b**) of the neat PBAT and PBAT-LDH (5 wt %) nanocomposites (heating rate 20 K·min^−1^ under nitrogen flow).

To investigate the influence of IL-treated LDHs on the PBAT nanocomposite thermal behavior, TGA was performed. Thus, the TGA and DTG curves of PBAT and PBAT containing 2 and 5 wt % of untreated and treated LDHs are presented in Figure 4 and Figure 5.

In all cases, the incorporation of pristine LDH or IL-modified LDH denoted LDH-349, LDH-351 and LDH-104 led to a decrease in the neat PBAT degradation temperature of about 10–15 °C. According to the literature, this phenomenon is attributed to the presence of water molecules in the LDH layers, leading to an acceleration of the PBAT degradation [41]. In fact, since LDH-ILs were introduced into the PBAT matrix without being previously dried and that the processing of the nanocomposites required a temperature of 160 °C, the presence of water in the LDHs can explain this phenomenon. Our previous work confirms these assumptions in which a weight loss between 5 and 7 wt % corresponding to the loss of physisorbed water between LDH layers is observed for LDH-349, LDH-351, and LDH-104, respectively [9]. In fact, many authors have also observed the same trend in polymer nanocomposites based on PLA and LDH [41,47]. Nevertheless, deeper investigation is still required. In summary, the incorporation of organically modified LDHs has only a slight influence on the thermal stability of the PBAT matrix. 

**Figure 6:**
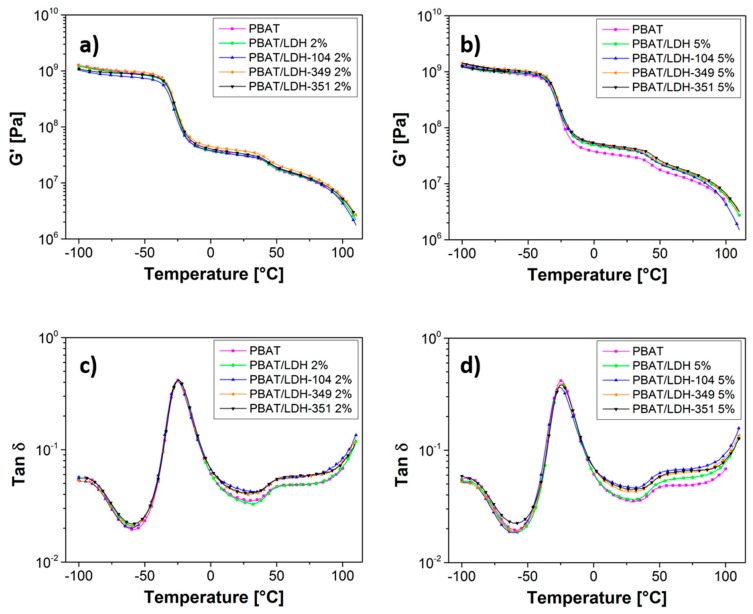
Storage moduli G’ (**a**,**b**), main relaxation peak of PBAT and the resulting nanocomposites evidenced on tan δ diagrams (**c**,**d**) recorded at 1 Hz.

Secondly, the impact of treated LDHs on the thermo-mechanical properties of PBAT was investigated by Dynamic Mechanical Analysis (DMA). The storage moduli G’ and the main relaxation peak evaluated as the maximum of tan δ peak are displayed in Figure 6. 

In all cases, a main relaxation peak at −24.9 °C and a shoulder peak at 55 °C were highlighted by DMA corresponding well with the literature [48]. Thus, Nayak et al. [48] demonstrated that the first transition corresponds to the motion of the polybutylene adipate unit while the second transition is attributed to the terephthalate unit. Then, the addition of pristine LDH as well as IL-treated LDH in amounts of 2 wt % or 5 wt % did not influence the temperature of these two relaxations. 

**Figure 7:**
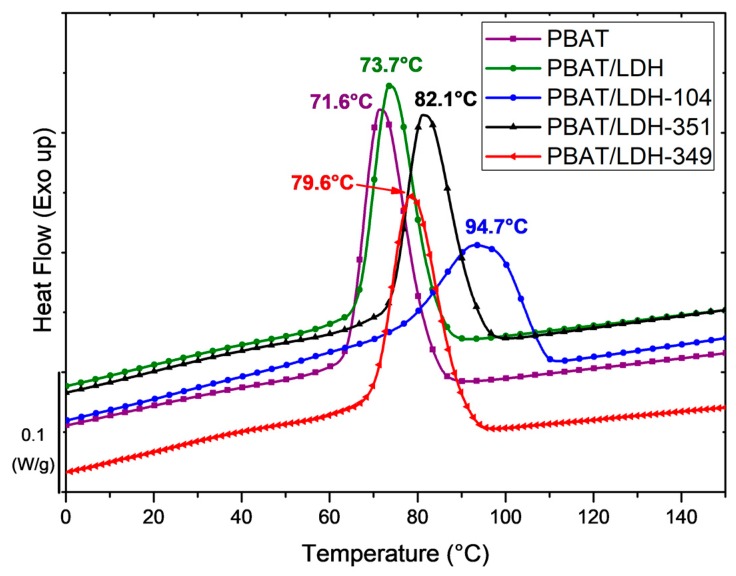
Differential scanning calorimetry (DSC) curves showing the crystallization temperatures T_c_ of neat PBAT and PBAT containing 5 wt % of untreated LDH and LDH-ILs.

As the DSC data showed no influence of the LDH-ILs on the glass transition temperature T_g_ and melting temperature T_m_ of the PBAT nanocomposites, the differential scanning calorimetry (DSC) curves highlighting the crystallization temperatures T_c_ of the PBAT and the resulting nanocomposites containing 5 wt % of LDH and LDH-ILs are represented in Figure 7. 

In the case of neat PBAT, a glass transition temperature of −34.6 °C and a melting temperature of 123 °C were obtained, which is in agreement with the literature [46,49]. The incorporation of only 2 wt % or 5 wt % of treated-LDHs showed no influence on the T_g_ or T_m_ of PBAT. However, differences can be observed for the crystallization temperatures. Whatever the amount of untreated LDH introduced into the PBAT matrix, no influence on the crystallization temperature (73 °C) was observed. These results are similar to those observed by Xie et al. which also showed that the incorporation of LDH containing nitrate ions in a PBAT matrix led to a decrease in the crystallization temperature [50]. In fact, this phenomenon can be attributed to the poor dispersion (Figure 2a) of the pristine LDH in the polymer thus impeding the crystallization of PBAT. Conversely, the use of LDH-ILs induced significant increases of T_c_ from 73 °C to 95 °C. Thus, crystallization temperatures of 80 °C, 82 °C, and 95 °C were obtained for LDH-349, LDH-351, and LDH-104, respectively. These results can be explained by the good dispersion of LDH-ILs in the PBAT matrix (Figure 2) inducing a heterogeneous nucleation effect as well as a lamellar ordering effect in the polymer matrix [50,51]. Thus, these DSC data confirm the presence of phosphonium IL at the surface of LDHs leading to a better affinity between LDH and PBAT. In addition, the good distribution of LDH-104 combined with the formation of IL104 ionic clusters (see Figure 2b) may explain the T_c_ value of 95 °C.

In summary, the presence of physisorbed and intercalated phosphonium ILs plays a key role on the compatibilization between the polymer matrix and the hydrotalcites.

#### 2.2.3. Mechanical Properties

In order to reveal the impact of the modified LDHs denoted LDH-104, LDH-349, and LDH-351 on the mechanical performances of the PBAT matrix, uniaxial tensile properties were performed and the fracture properties and moduli are summarized in Table 1.

Independently of the amount (2 or 5 wt %) of untreated LDH incorporated into PBAT, no significant influence was observed on the final mechanical performances of the polymer matrix. These results are in agreement with the literature where different authors have demonstrated that the addition of unmodified LDH had no impact on the mechanical properties of the matrix [37,50]. Nevertheless, the use of phosphonium ILs modified LDHs led to different behaviors depending on the IL’s chemical nature. Thus, IL351 and IL349 led to increases in the Young’s modulus of the order of 20–30% for only 2 wt % of fillers and 30–50% when 5 wt % of modified LDHs are used. In addition, only slight decreases of the fracture behavior are obtained (of the order of 10–15%). These results can be explained by their respective morphologies (Figure 2) where a good dispersion was observed but also due to the good affinity between LDH-ILs and PBAT. The results are promising compared to those reported in the literature [50,52]. In fact, different authors have highlighted that the organic modification of LDH by stearic acid, lauryl alcohol phosphoric acid ester potassium, dodecylsulfate or decanoate counter anions has no effect on the mechanical behavior of polyester nanocomposites based on PLA [50,52]. For example, Xie et al. demonstrated an increase of only 12.5% of the elongation at break with 7.5% of the tensile strength [50]. In the case of IL104, increases in the strain at break without reducing the Young Modulus of the neat PBAT are observed going from 511% to 570% and 940% when 2 wt % and 5 wt % were used, respectively. This phenomenon can be attributed to the well dispersed ionic clusters (IL104, Figure 2), as observed when ionomers or ILs are used [53,54].

In conclusion, the use of phosphonium ILs as interfacial agents of LDH led to a significant improvement of the mechanical performances of PBAT matrix, which offers potential advances in the field of compostable films or food packaging applications.

#### 2.2.4. Influence of Modified-LDHs on the Gas Transport Properties of PBAT Matrix

The LDH surface treatment influence, as well as the previously obtained different morphologies, were investigated by the permeability coefficients of various gases in neat PBAT and PBAT nanocomposites. The permeability coefficients of H_2_, O_2_, N_2_, CO_2_, H_2_O and the ideal selectivities of selected gas pairs are presented in Table 2. Diffusion and solubility coefficients and their corresponding ideal selectivities are presented in the supplementary material (Tables S1 and S2). 

In all cases, the permeability coefficients of H_2_, O_2_, N_2_, CO_2_, H_2_O increases in the following order:

N_2_ < O_2_ < H_2_ < CO_2_ << H_2_O

Rigid polymer structures of glassy polymers promote molecules with higher diffusion coefficients and because *D*(H_2_) >> *D*(CO_2_) permeability coefficients are usually larger than those of CO_2_ [55]. In this case, flexible blocks in PBAT and relatively high polarity led to strong interaction between PBAT and CO_2_ as well as the polymer matrix being above its T_g_. In other words CO_2_ exhibits significant interactions with the polymer which means high solubility (sorption coefficients) in the polymer and this phenomenon causes CO_2_ to permeate significantly faster than H_2_ (Tables S1 and S2 in supplementary material). These results are in agreement with the literature where different authors have highlighted this phenomenon which can be explained by the interactions between CO_2_ (polar molecule) with the ester groups of PBAT [49,56]. In addition, due to this excellent affinity, other research groups investigated the foaming of PBAT under supercritical CO_2_ [57]. Nitrogen, on the other hand, interacts with the polymer very slightly. Thus, we can observe relatively high selectivities for the CO_2_/N_2_ gas pair (Table 2) which makes these materials potentially suitable for CO_2_/N_2_ separations. Other gas pairs exhibited only moderate values comparable with most general polymers. 

From transport data in Table 2 we can surprisingly see a slight influence of all untreated and treated fillers on both permeabilities and selectivities. In fact a slight decrease in permeabilities can be observed with addition of untreated and treated LDHs. From the morphological point of view most of the gas transport occurs in the neat polymer matrix due to low filler contents, especially in the case of untreated LDHs which creates agglomerates. Agglomerates (seen in Figure 2a) themselves can contain empty spaces where the gas can diffuse freely and thus these spaces promote permeabilities and maintain selectivity, which is determined mostly by the polymer matrix itself. On the other hand treated LDHs tends to disperse well in the polymer matrix, as well as to exfoliate some of its layers which can act as barriers for the gas diffusion. Exfoliation can also make intercalated IL more accessible to interactions with diffusing gases. In our case no such effects played a significant role except for the water vapor (see below). This is in agreement with the theory of transport properties of polymeric materials filled with impermeable or low permeability particles. Filler particles, especially layered ones, partially block pathways for gas molecules penetrating in free volume among the PBAT macromolecules [58,59]. As XRD data have shown the basal spacing of the LDH is not much influenced by the anion type, the same could be concluded for gas permeabilities [9].

Moreover, even stronger interactions between gas and polymer are observed for water vapor. Highly polar water molecules with small molecule dimensions cause several orders of magnitude of higher water vapor permeabilities in PBAT materials compared with N_2_ and O_2_, which are the main components of air. However, the incorporation of modified LDHs into the PBAT matrix led to significant decreases in water vapor permeability varying with function of the chemical nature of the phosphonium LIs as well as the morphologies previously generated (see Figure 2). Indeed, LDH-104 and LDH-349 led to the most significant decreases in water vapor permeability coefficient. These results can be explained by their respective homogeneous semi-exfoliated morphologies. In the case of PBAT/LDH-104, a decrease of 50% is obtained independently of the amount of modified-LDH used. This is a result of the excellent dispersion of the lamellar fillers as well as of the dispersion of ionic clusters Higher surface of highly polar and well dispersed semi-exfoliated particles create more sorption sites, where the water molecules could sorb resulting in a large number of strong interactions slowing down the water molecules’ diffusion through the membrane. Water vapor measurements were performed at water partial pressures close to the saturated one—this means completely non-Fickean diffusion so the diffusion coefficient *D* and the solubility *S* parameters in Tables S1 and S2 are only apparent. Concerning PBAT/LDH-351, presenting an intercalated morphology combined with the presence of few aggregates, this decrease is of the order of 30%. On the other hand, the poor dispersion of unmodified LDH into the PBAT matrix resulted in a very slight decrease of only 10%. 

In summary, the presence of IL modified-LDHs has no measurable effect on gas transport due to similar diffusion and sorption coefficients of gases in ILs and PBAT matrix, but a significant influence on the water vapor permeation has been highlighted. Thus, these nanocomposites can be good candidates for food packaging applications. 

## 3. Materials and Methods 

An Layered Double Hydroxide LDH (aluminum magnesium hydroxy carbonate) denoted PURAL^®^ MG 63 HT) was chosen as pristine anionic clay and was provided by Sasol Performance Chemicals (Hamburg, Germany). PBAT used in this study was supplied by BASF (Ludwigshafen, Germany) under the trade name of Ecoflex. The ILs denoted IL104, IL351, and IL349 based on tributyltetradecylphosphonium cation associated with phosphinate, carboxylate and phosphate counter anions were kindly provided by Cytec Industries Inc. (Thorold, ON, Canada) 

Organic modification of LDH: First, the pristine LDH was heated for 24 h at 500 °C to obtain calcined LDH. Then, based on the Anionic Exchange Capacity (AEC = 3.35 meq/g) of the LDH used [9,37,60], LDH and 2 AEC of phosphonium ILs were dispersed in 200 mL of deionized water/tetrahydrofuran mixture (300/100 mL). After, the suspensions were stirred and mixed at 60 °C over 24 h. The resulting precipitate was filtered and washed 5 times with THF. The residual solvent was removed by evaporation under vacuum and finally, the treated LDH was dried overnight at 80°C. The phosphonium ILs used for the anionic exchange and the following abbreviations used to designate the various treated-LDHs are summarized in Table 3. 

Nanocomposites based on PBAT/organically treated LDHs (2 and 5 wt %) i.e., PBAT/LDH, PBAT/LDH-104, PBAT/LDH-351, PBAT/LDH-349 were processed using a 15 g-capacity DSM micro-extruder (DSM Research, Heerlen, The Netherlands) with co-rotating screws (Lenght/Diameter L/D ratio of 18). The mixture was sheared over 3 min with a 100 rpm speed at 160 °C and injected into a 10 cm^3^ mold at 30 °C to obtain dumbbell-shaped specimens. 

Thermogravimetric analysis (TGA) of ILs, untreated and treated-LDH and nanocomposites were performed on a Q500 thermogravimetric analyzer (TA instruments, New Castle, DE, USA). The samples were heated from room temperature to 600 °C at a rate of 20 K·min^−1^ under nitrogen flow.

Differential Scanning Calorimetry measurements *(DSC)* of PBAT and the resulting nanocomposites were performed on a Q20 (TA instruments) from −60 °C to 180 °C. The samples were kept for 1 min at 180 °C to erase the thermal history before being heated or cooled at a rate of 10 K·min^−1^ under nitrogen flow of 50 mL·min^−1^. The crystallinity was calculated with the heat of fusion for PBAT of 114 J/g [61].

Wide-angle X-ray diffraction spectra (WAXD) were collected on a D8 Advance X-ray diffractometer (Bruker, Billerica, MA, USA) at the Henri Longchambon diffractometry center. A bent quartz monochromator was used to select the Cu K_α1_ radiation (λ = 0.15406 nm) and run under operating conditions of 45 mA and 33 kV in Bragg-Brentano geometry. The angle range scanned is 1–10° 2θ for the modified clays and for the nanocomposite materials.

Transmission electron microscopy (*TEM*) was carried out at the Center of Microstructures (University of Lyon, France) on a Philips CM 120 field emission scanning electron microscope (Philips, Amsterdam, The Netherlands) with an accelerating voltage of 80 kV. The samples were cut using an ultramicrotome (Leica, Weitzlar, Germany) equipped with a diamond knife, to obtain 60 nm-thick ultrathin sections. Then, the sections were set on copper grids.

Uniaxial Tensile Tests were carried out on a MTS 2/M electromechanical testing system (MTS, Eden Prairie, MN, USA) at 22 °C ± 1 °C and 50 ± 5% relative humidity at crosshead speed of 50 mm·min^−1^.

Dynamic mechanical analyse*s* were performed on ARES G2 rheometer (TA Instruments). The temperature dependence of the complex shear modulus of rectangular samples (dimension: 20 × 5 × 1.5 mm^3^) was measured by oscillatory shear deformation at a frequency of 1 Hz and a heating rate of 3 °C·min^−1^ in a temperature range of −100 to +100 °C. The temperature of a relaxation was evaluated as the maximum of tan δ peak.

Gas transport properties of PBAT with various content of LDH were examined by the time-lag permeation method [62]. Samples in the form of thin films (prepared in a hot-press at 160 °C) were inserted into a membrane cell which was then placed into a permeation apparatus. The sample was then exposed to high vacuum (10–4 mbar) and temperature 45 °C for 12 h. After evacuation the temperature was set on 30 °C. Then 2–3 samples of each membrane were measured. Feed pressure *p_i_* was 1.5 bar. The permeability coefficient *P* was determined from the increase of the permeate pressure Δ*P_p_* per time interval Δ*t* in a calibrated volume *V_p_* of the product part during the steady state of permeation. For calculation of permeability coefficient, the following formula was used:

(1) $$P=\frac{\Delta p_{p}}{\Delta t}\times \frac{V_{p}l}{Ap_{i}}\times \frac{1}{RT}$$

where *l* is the membrane thickness, *p_i_* feed pressure, *A* the area, *T* the temperature, and *R* the gas constant. Relative standard deviations (SD) of Δ*P_p_* and Δ*t* were lower than 0.3% (given by the 10 mbar MKS Barratron pressure transducer precision). Relative SD of membrane thickness measurement was 1%, relative SD of calibrated volume was lower than 0.5%, and relative SD of feed pressure was 0.3%. Therefore *P* values had the relative SD 2.4%, very low values of *P* (below 0.01 Barrers) had relative SD up to 15%, due to lower (nonlinear) precision of the MKS Baratron at very low pressures. 

Gas diffusivities were estimated from the time-lag data, using the relation:

(2) $$D\;=\;\frac{l^{2}}{6\theta }$$

where *D* is the diffusion coefficient, *l* is the film thickness and *θ* is the time-lag. Relative standard deviation of diffusion coefficients was 4%. A precision of 0.1 s for the time-lag determination allowed the determination of the diffusion coefficients of hydrogen with relative standard deviation of 8%. Apparent solubility coefficients were calculated using the following equation:

(3) S=P/D

The overall selectivity of a polymer membrane for a pair of gases *i* and *j* is commonly expressed in terms of an ideal separation factor, α*ij*, defined by the following relation:

(4) $$\alpha_{ij}=\frac{P_{i}}{P_{j}}=\frac{S_{i}}{S_{j}}\times \frac{D_{i}}{D_{j}}$$

where *P_i_* and *P_j_* are pure gas permeabilities, *D_i_*/*D_j_* is the diffusion selectivity and *S_i_*/*S_j_* is the solubility selectivity.

## 4. Conclusions

In this work, phosphonium ILs were used as LDH interfacial agents to prepare thermally stable LDHs by the extrusion process. Firstly, these modified-LDHs were characterized by TGA and X-ray diffraction, highlighting the intercalation of the counter anions among the LDH layers as well as the presence of physisorbed ILs on the LDHs surface. Then, different amounts (2 and 5 wt %) of these organically modified LDHs were introduced into a PBAT matrix by melt extrusion, resulting in partially exfoliated morphologies. Thus, a mechanical performances increase (20% to 50% of the Young’s modulus) combined with a water barrier properties increase (30% to 50%) was obtained for all IL-modified-LDH based nanocomposites. These results demonstrated the influence the surfactant’s chemical nature, particularly the counter anion, has on the morphologies as well as on the final properties of the PBAT matrix. Altogether, these PBAT based nanocomposites open new possibilities for the field of compostable films and food packaging applications.

## Figures and Tables

**Table 1:** Mechanical performances of neat poly(butylene adipate-co-terephthalate) (PBAT) and the resulting nanocomposites.

Nomenclature	Young Modulus (Mpa)	Stress (Mpa)	Strain at Break (%)
PBAT	47 ± 1	24 ± 1	511 ± 17
PBAT/LDH	56 ± 1	24 ± 1	577 ± 18
PBAT/LDH-349 2%	62 ± 1	22 ± 1	462 ± 30
PBAT/LDH-349 5%	72 ± 2	22 ± 1	400 ± 13
PBAT/LDH-104 2%	52 ± 2	22 ± 1	567 ± 7
PBAT/LDH-104 5%	50 ± 1	22 ± 1	940 ± 10
PBAT/LDH-351 2%	57 ± 2	21 ± 1	455 ± 30
PBAT/LDH-351 5%	61 ± 1	20 ± 2	440 ± 15

**Table 2:** Dependence of gas and water vapor permeability coefficients and corresponding ideal selectivities in PBAT polymer materials containing different amount of pristine and modified-LDHs.

Materials	Permeability Coefficient (Barrer) ^a^					Ideal Selectivity				
	H_2_	O_2_	N_2_	CO_2_	H_2_O	H_2_/N_2_	H_2_O/O_2_	O_2_/N_2_	CO_2_/H_2_	CO_2_/N_2_
PBAT	4.92	1.22	0.33	12.5	2580	15.1	2110	3.7	2.5	38.3
PBAT/LDH 2%	4.53	1.04	0.31	11.0	1970	14.8	1900	3.4	2.4	36.1
PBAT/LDH 5%	4.38	0.95	0.31	10.0	2210	14.3	2320	3.1	2.3	32.7
PBAT/LDH-349 2%	4.01	0.95	0.29	9.9	1630	13.8	1730	3.3	2.5	33.9
PBAT/LDH-104 2%	3.74	0.93	0.27	9.5	1360	13.7	1470	3.4	2.5	34.8
PBAT/LDH-349 5%	4.97	1.08	0.34	11.7	1400	14.6	1300	3.2	2.4	34.5
PBAT/LDH-351 5%	4.20	1.00	0.28	10.1	1520	15.2	1520	3.6	2.4	36.5
PBAT/LDH-104 5%	4.27	1.05	0.32	10.4	1710	13.2	1630	3.2	2.4	32.1

^a^ Barrer = 1 × 10^−10^ cm^3^ (STP) cm/(cm^2^ s cm Hg) = 3.3539 × 10^−16^ mol s^−1^ m^−1^ Pa^−1^.

**Table 3:** Designation of ionic liquids (ILs) used for the surface treatment of layered double hydroxide (LDH).

Ionic Liquid	Chemical Structure	Designation
Trihexyl(tetradecyl)phosphonium bis(2,4,4-trimethylpentyl)phosphinate	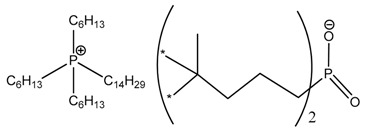	LDH-104
Trihexyl(tetradecyl)phosphonium 2-ethylhexanoate	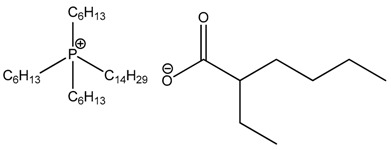	LDH-351
Trihexyl(tetradecyl)phosphonium bis(2-ethylhexyl)phosphate	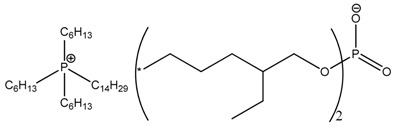	LDH-349

## Supplementary Materials

The dependence of gas and water vapor diffusion and of gas and water vapor solubility coefficients in PBAT polymer materials containing different amount of pristine and modified-LDHs following are available online at http://www.mdpi.com/2079-4991/7/10/297/s1, Table S1, Dependence of gas and water vapor diffusion coefficients in PBAT polymer materials containing different amount of pristine and modified-LDHs, Table S2: Dependence of gas and water vapor solubility coefficients in PBAT polymer materials containing different amounts of pristine and modified-LDHs.
