# Supplementary material for: Ionic Liquids as Surfactants for Layered Double Hydroxide Fillers: Effect on the Final Properties of Poly(Butylene Adipate-Co-Terephthalate)
Source: Nanomaterials (Basel). 2017 Sep 28;7(10):297. doi: 10.3390/nano7100297 (PMC5666462; doi:10.3390/nano7100297)
Supplement: Supplementary file 1 [file nanomaterials-07-00297-s001.pdf]

Ionic Liquids as Surfactants for Layered Double Hydroxide Fillers: Effect on the Final Properties of Poly(Butylene Adipate-Co-Terephthalate)

Sébastien Livi [1,*], Luanda Chaves Lins [1,*], Jakub Peter [2], Hynek Benes [2], Jana Kredatusova [2], Ricardo K. Donato [3] and Sébastien Pruvost [1]

[1] IMP Ingénierie des Matériaux Polymères, UMR CNRS 5223, INSA Lyon, Université de Lyon, F-69621 Villeurbanne, France
[2] Institute of Macromolecular Chemistry AS CR, v.v.i., Heyrovsky Sq. 2, 162 06 Prague 6, Czech Republic; peter@imc.cas.cz (J.P.); benesh@imc.cas.cz (H.B.); kredatusova@imc.cas.cz (J.K.)
[3] MackGraphe—Graphene and Nanomaterials Research Center, Mackenzie Presbyterian University, Rua da Consolação 896, São Paulo 01302-907, Brazil; ricardo.donato@mackenzie.br
* Correspondence: sebastien.livi@insa-lyon.fr (S.L.); Tel.: +33-472-438-291 (S.L.); luandaqmc@gmail.com (L.C.L.)

Table S1: Dependence of gas and water vapor diffusion coefficients in poly(butylene adipate-co-terephthalate) (PBAT) polymer materials containing different amount of pristine and organically treated- layered double hydroxides (LDHs)

	Diffusion coefficient $D \times 10^{12}$ (m^2/s)					Diffusivity selectivity				
Material	H_2	O_2	N_2	CO_2	H_2O	H_2/N_2	O_2/H_2O	O_2/N_2	H_2/CO_2	CO_2/N_2
PBAT	360	34	17	11.3	5.7	21.5	6.0	2.1	31.9	0.7
PBAT-LDH 5%	310	34	25	11.3	4.1	12.3	8.4	1.4	27.5	0.4
PBAT-LDH 10%	300	27	25	9.0	3.5	11.9	7.7	1.1	33.4	0.4
PBAT/LDH-349 5 %	-	26	17	9.0	3.3	-	7.9	1.5	-	0.5
PBAT/LDH-104 5 %	-	24	19	7.8	2.3	-	10.3	1.3	-	0.4
PBAT/LDH-349 10 %	320	28	14	10.8	2.2	0.0	12.7	0.0	0.7	0.0
PBAT/LDH-351 10 %	328	30	16	10.0	2.6	20.8	11.7	1.9	32.7	0.6
PBAT/LDH-104 10 %	303	28	28	9.5	2.1	11.0	13.6	1.0	31.8	0.3

Table S2: Dependence of gas and water vapor solubility coefficients in PBAT polymer materials containing different amount of pristine and organically treated-LDHs

	Solubility coefficient $S \times 10^6$ (mol.m3/Pa)					Solubility selectivity				
Material	H_2	O_2	N_2	CO_2	H_2O	H_2/N_2	H_2O/O_2	O_2/N_2	CO_2/H_2	CO_2/N_2
PBAT	4.6	11.9	6.54	372	153 000	0.7	12900	1.8	81.2	56.9
PBAT-LDH 5%	4.9	10.1	4.06	329	162 000	1.2	16100	2.5	67.0	80.9
PBAT-LDH 10%	4.9	11.9	4.06	373	212 000	1.2	17800	2.9	76.2	91.9
PBAT/LDH-349 5 %	-	12.4	5.82	369	169 000	-	13700	2.1	-	63.5
PBAT/LDH-104 5 %	-	13.2	4.85	407	199 000	-	15200	2.7	-	83.8
PBAT/LDH-349 10 %	5.2	13.0	8.16	365	214 000	0.6	16600	1.6	69.9	44.7
PBAT/LDH-351 10 %	4.3	11.1	5.90	338	197 000	0.7	17800	1.9	78.6	57.2
PBAT/LDH-104 10 %	4.7	12.6	3.95	367	280 000	1.2	22300	3.2	77.5	93.0
